# Resection of the primary tumor improves survival in patients with gastro‐entero‐pancreatic neuroendocrine neoplasms with liver metastases: A SEER‐based analysis

**DOI:** 10.1002/cam4.2431

**Published:** 2019-07-22

**Authors:** Mengzhen Zheng, Yan Li, Tong Li, Lianfeng Zhang, Lin Zhou

**Affiliations:** ^1^ Department of Gastroenterology The First Affiliated Hospital of Zhengzhou University Zhengzhou China; ^2^ Xinxiang Medical University Xinxiang China

**Keywords:** liver metastases, neuroendocrine neoplasms, SEER, survival

## Abstract

Patients who suffer from gastro‐entero‐pancreatic neuroendocrine neoplasms (GEP‐NENs) often present with liver metastatic disease. The efficacy of primary tumor resection (PTR) for these patients remains controversial due to the relatively heterogeneous behavior of the primary tumor and the lack of clinical evidence. In this series, GEP‐NEN patients with liver metastases (LM) were selected from the Surveillance, Epidemiology, and End Results database between 2010 and 2015. A logistic regression model was used to analyze variables that were associated with PTR. A Cox proportional hazards model was used to identify independent prognostic risk factors. In total, 1547 patients were enrolled in our study, including 897 patients who underwent PTR. Resection of the primary tumor was associated with prolonged survival in all patients (5‐year overall survival (OS) rates: 57.0% vs 15.4%, *P* < .001), and improved 5‐year OS rates were observed in patients with gastric, small intestinal, colorectal, and pancreatic subtypes (39.7%, 73.3%, 24.6%, and 59.7%, respectively). On the multivariate analysis, PTR was an independent prognostic factor of prolonged OS (HR = 0.48, 95% CI: 0.39‐0.59, *P* < .001). Patients with a young age (≤60 years), small intestinal or colorectal NENs, a large primary tumor, lymph node (LN) metastases, and high tumor differentiation were more likely to undergo PTR. However, patients with colorectal NENs or a large primary tumor (≥4 cm) were at an increased risk of death independently in the PTR subgroup. The risk factors for OS also included old age, gastric tumor location, and poor differentiation. In conclusion, although PTR prolonged OS in all GEP‐NEN patients presenting with LM, surgical recipients should be considered carefully. Age, primary tumor site, size, and differentiation might help surgeons identify patients who could benefit from PTR.

## 1 INTRODUCTION

Neuroendocrine neoplasms (NENs) are a relatively rare group of tumors. They used to be defined as “carcinoid” because of their heterogeneous and indolent nature. However, the incidence of NENs has significantly increased from 1.09 per 100 000 persons in 1973 to 6.98 per 100 000 persons in 2012.^1^ NENs occur in a variety of sites throughout the body, and more than half are gastrointestinal or pancreatic neuroendocrine neoplasms (GEP‐NENs).^2^ Although GEP‐NENs are slow‐growing malignancies, up to 40%‐45% of patients are initially diagnosed with distant metastases, which frequently present with liver metastases (LM).^3^ Since these tumors are capable of producing hormones that cause severe hormonal syndromes, patients with LM often have inferior quality of life. In addition, patients will eventually die from tumor progression, gastrointestinal obstruction, and liver failure.

Although multiple treatment options for liver metastatic disease consist of locoregional and/or liver surgery, ablative therapies, and systemic chemotherapy, the only potentially curative treatment approach for patients is complete resection of both the primary and metastatic tumors. Indeed, only approximately 20% of patients who undergo tumor resection for curative intent are eligible.^4^ Even if curative resection cannot be implemented, there is still potential benefit, as shown when 80%‐90% of the tumor burden can be reduced.^5^ Previous studies have demonstrated that both curative and debulking hepatic resection surgeries benefit survival.^6^ However, the debate still remains about whether primary tumor resection (PTR) benefits the outcome of patients because of its heterogeneous nature. Recently, a retrospective study by Tierney et al showed significantly prolonged survival of patients with metastatic disease who underwent PTR.^7^ Nevertheless, this study did not make a distinction between LM and other sites of metastases, and several studies that support PTR were limited by their small sample size and the analysis of a single primary site.^8^, ^9^

Given that the liver is the predominant metastatic site, our study focused on determining the efficiency of PTR against liver metastatic NENs using a large cohort from the Surveillance, Epidemiology, and End Results (SEER) database. Furthermore, we also aimed to confirm the potential prognostic factors that might provide more robust evidence for surgeons to make determinations for selecting patients.

## 2 METHODS

### 2.1 Data collection

Patients were identified from the SEER database from 2010 to 2015 using SEER*Stat software (version 8.3.5, National Center Institute). All patient data were extracted from the SEER database according to the International Classification of Diseases for Oncology, third edition (ICD‐O‐3) primary site code (stomach C16.0 to C16.9, intestine C17.0 to C17.9 and C24.1, appendix C18.1, colon C18.0 and C18.2 to C18.9, rectum C19.9 and C20.9, and pancreas C25.0 to C25.9) and histology code (8013/3, 8150/3, 8151/3, 8152/3, 8153/3, 8155/3, 8156/3, 8240/2, 8240/3, 8241/3, 8242/3, 8243/3, 8244/3, 8245/3, 8246/2, 8246/3, 8247/2, 8247/3, 8248/3, and 8249/3). The inclusion criteria were as follows: (a) GEP‐NENs with a positive diagnosis from histology or exfoliative cytology; and (b) the presence of LM without other metastatic organs. The exclusion criteria were as follows: (a) more than one primary tumor; (b) incomplete follow‐up data; and (c) the metastatic tumor was resected. Only patients diagnosed between 2010 and 2015 were enrolled because the SEER database has provided information on the specific locations of metastatic tumors since 2010.

The following clinical data were retrieved and analyzed to determine whether they are associated with prognosis: age, sex, race, primary tumor site, tumor differentiation, primary tumor size, lymph node (LN) status, surgery, and follow‐up information. Overall survival (OS) was defined as the survival time from a positive diagnosis to death. The patients were analyzed in two subgroups (resection and nonresection). In addition, we did not extract the WHO 2010 classification of tumors from the SEER database, which was replaced by tumor differentiation (from I to IV).

### 2.2 Statistical analysis

Student's *t* test and the Chi‐squared test (or Fisher's exact test) were performed in two subgroups depending on the categorical and ordinal variables. We also used a logistic regression analysis to identify variables that might be associated with recipients of PTR. OS was analyzed by Kaplan‐Meier with the log‐rank test based on different primary tumor subtypes. Univariate and multivariate analyses were carried out using the Cox proportional hazards model to identify independent prognostic risk factors. All statistical analyses were conducted using GraphPad Prism (version 7, GraphPad Software Inc), and statistical significance was considered when the *P* value was <.05.

## 3 RESULTS

### 3.1 Patient and tumor characteristics

In total, 1547 patients were selected from the SEER database between 2010 and 2015. The characteristics of the patients and tumors are summarized in Table 1. Among these patients, 897 (58.0%) underwent PTR and 650 (42.0%) did not. The patients' ages ranged from 10 to 85 years, and the median age was 57.6 years. The majority of patients were White (N = 1231, 79.6%). Primary tumors located in the small intestine (N = 556, 35.9%) and pancreas (N = 501, 32.4%) were more likely to metastasize to the liver, followed by the colorectum (N = 391, 25.3%) and stomach (N = 99, 6.4%). Additionally, to gain insight into the correlation between clinicopathological factors and PTR, a logistic regression was performed. Compared to the nonresection group, an intestinal or a colorectal primary tumor site, larger primary tumor, LN metastases, and higher differentiation (well and moderately differentiated) were associated with a higher likelihood of undergoing PTR (*P* < .05). The results are listed in Table 2.

**Table 1:** Characteristics of GEP‐NENs patients with liver metastases in SEER database (N = 1547)

Characteristics	Total	Resection	Nonresection	*P* value
	N = 1547	N = 897	N = 650	
Gender				.005
Male	842	461	381	
Female	705	436	269	
Age at diagnosis				.007
<60	767	471	296	
≥60	780	426	354	
Race				.002
White	1231	738	493	
Black	208	112	96	
Other*	108	47	61	
Primary tumor site				<0.001
Stomach	99	19	80	
Small intestine	556	447	79	
Colorectum	391	233	158	
Pancreas	501	168	333	
Primary tumor size				<.001
≤2 cm	278	245	33	
2‐4 cm	428	314	114	
≥4 cm	602	307	295	
Unknown	239	31	208	
LN metastases				<.001
Yes	958	717	241	
No	464	167	297	
Unknown	125	13	112	
Differentiation				<.001
Well differentiated	726	505	221	
Moderately differentiated	310	204	106	
Poorly differentiated	352	122	230	
Undifferentiated	159	66	93	

Note

Other*: American Indian/AK Native, Asian/Pacific Islander, unknown.

Abbreviation: LN: lymph nodes.

**Table 2:** Multivariate analysis of factors associated with recipients of PTR

Variables	Odds radio	95% CI	*P* value
Gender			
Male	1.00		
Female	1.30	0.98‐1.73	.073
Age at diagnosis			
<60	1.00		
≥60	0.86	0.65‐1.15	.303
Race			
White	1.00		
Black	0.67	0.44‐1.03	.069
Other*	0.70	0.41‐1.19	.190
Primary tumor site			
Stomach	1.00		
Small intestine	8.78	4.38‐17.62	<.001
Colorectum	4.16	2.18‐7.91	<.001
Pancreas	0.76	0.40‐1.45	.406
Primary tumor size			
≤2 cm	1.00		
2‐4 cm	34.10	17.80‐65.10	<.001
≥4 cm	18.50	10.80‐31.90	<.001
Unknown	12.30	7.20‐20.90	<.001
LN metastases			
Yes	1.00		
No	0.22	0.16‐0.29	<.001
Unknown	0.06	0.03‐0.12	<.001
Differentiation			
Well differentiated	1.00		
Moderately differentiated	1.13	0.76‐1.66	.551
Poorly differentiated	0.29	0.19‐0.43	<.001
Undifferentiated	0.29	0.17‐0.48	<.001

Note

Other*: American Indian/AK Native, Asian/Pacific Islander, unknown.

Abbreviations: LN: lymph nodes; CI: Confidence interval.

### 3.2 OS and prognostic factors

**Figure 1:**
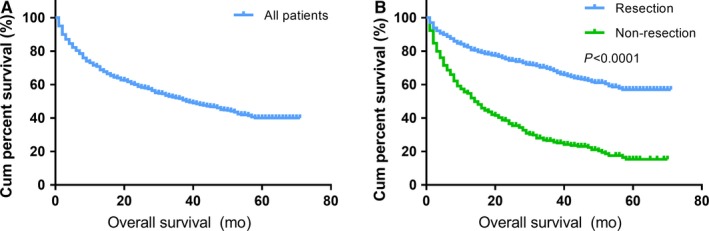
Kaplan‐Meier curves of OS in all patients. The median OS of all patients was 38 months (A). The median OS of the resection and nonresection subgroups was NR and 14 months, respectively (B). OS: overall survival; NR: not reached

**Figure 2:**
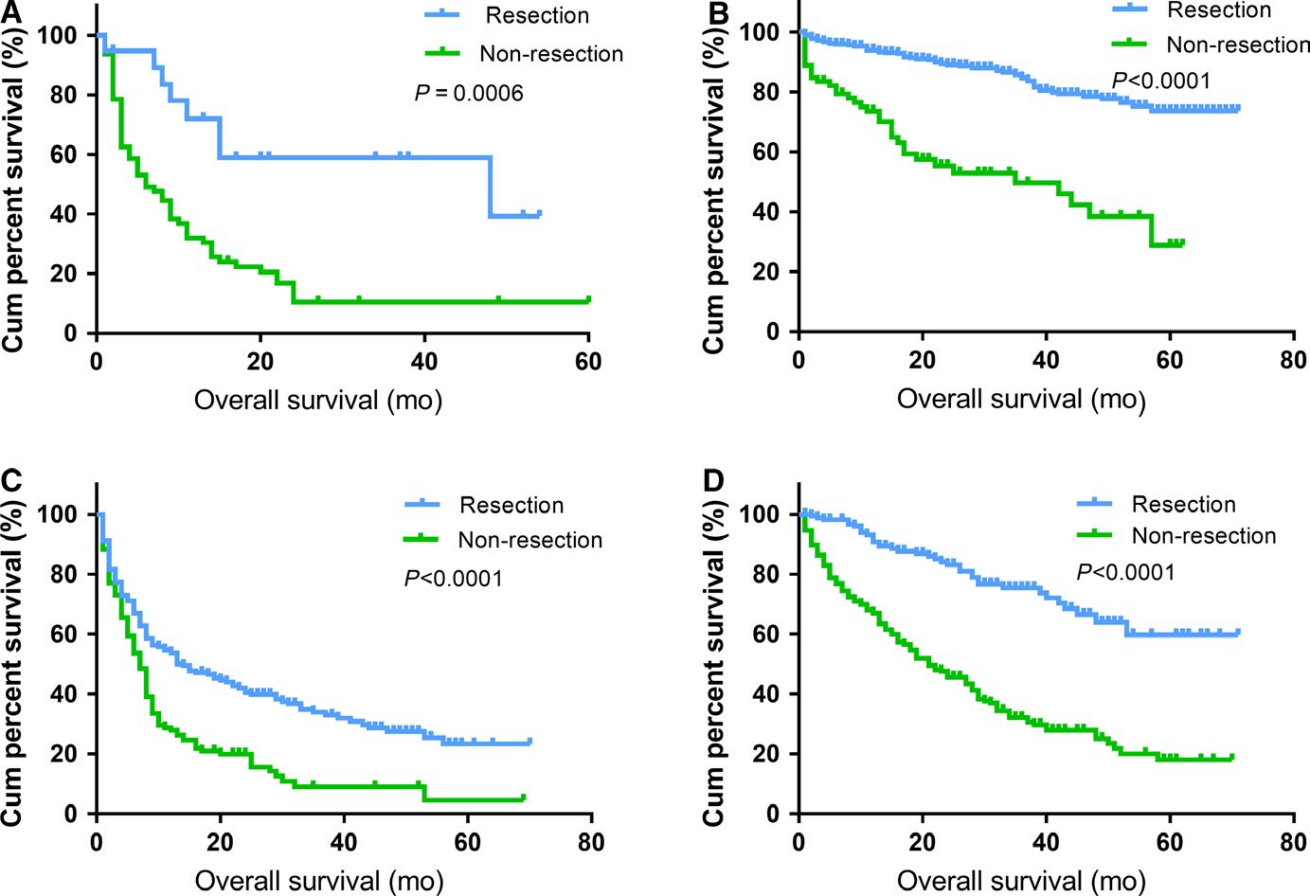
Kaplan‐Meier curves of OS in patients according to tumor subtype. PTR prolonged the median OS of patients with gastric NENs (48 months vs 6 months; A), small intestinal NENs (NR vs 42 months; B), colorectal NENs (13 months vs 7 months; C), and pancreatic NENs (NR vs 21 months; D). OS: overall survival; NENs: neuroendocrine neoplasms; NR: not reached

The median follow‐up time was 15 months (range from 1 to 71 months). There were 651 (42.1%) deaths recorded during the follow‐up period. As shown in Figure 1, the median OS for all patients was 38 months, with a 5‐year survival rate of 40.0%. The 5‐year OS rate for patients whose primary tumors were resected was 57.0%, while patients who did not undergo PTR had a 5‐year OS rate of 15.4%. The difference in median OS between the two subgroups was significant (not reached vs 14 months, *P* < .001). Moreover, patients were divided into four subtypes according to the primary tumor location, and OS curves are presented in Figure 2. The prolonged 5‐year OS rates of gastric, small intestinal, colorectal, and pancreatic NENs were detected after patients underwent PTR (39.7%, 73.3%, 24.6%, and 59.7%, respectively), which were significantly different from the patients who did not undergo PTR (10.5%, 29.9%, 4.7%, and 18.1%, respectively). A log‐rank test showed that survival differences between subgroups were significant in all subtypes (*P* < .01).

Furthermore, a univariate analysis was performed to identify the factors that might be associated with prolonged survival rates. Then, to identify the prognostic risk factors, a multivariate Cox regression was performed. The univariate analysis demonstrated that sex, age, primary tumor sites, primary tumor size, differentiation, and PTR were significant prognostic factors for patient survival (*P* < .05). In the multivariate analysis, we found that PTR was still an independent prognostic factor of prolonged OS (HR = 0.48, 95% CI: 0.39‐0.59, *P* < .001). In addition, factors including age, primary site, primary tumor size, and differentiation were also significantly associated with survival (Table 3).

**Table 3:** Univariate and multivariate survival analyses of overall survival in SEER database (N = 1547)

Characteristics	5‐year OS (%)	Univariate analysis		Multivariate analysis	
		HR (95% CI)	*P* value	HR (95% CI)	*P* value
Gender					
Male	36.2	1.00		1.00	
Female	44.5	0.79 (0.68‐0.92)	.003	0.83 (0.71‐0.98)	.025
Age at diagnosis					
<60	47.7	1.00		1.00	
≥60	32.3	1.60 (1.37‐1.87)	<.001	1.58 (1.35‐1.85)	<.001
Race					
White	39.1	1.00			
Black	45.0	1.03 (0.82‐1.28)	.829		
Other*	34.7	0.95 (0.69‐1.29)	.725		
Primary tumor site					
Stomach	15.6	1.00		1.00	
Small intestine	67.4	0.14 (0.10‐0.19)	<.001	0.52 (0.37‐0.74)	<.001
Colorectum	17.0	0.86 (0.66‐1.12)	.164	1.22 (0.92‐1.60)	.084
Pancreas	32.2	0.41 (0.31‐0.53)	<.001	0.64 (0.48‐0.85)	.006
Primary tumor size					
≤2 cm	68.2	1.00		1.00	
2‐4 cm	54.1	1.78 (1.28‐2.47)	.001	1.22 (0.87‐1.70)	.247
≥4 cm	26.8	4.18 (3.09‐5.65)	<.001	1.58 (1.14‐2.19)	.007
Unknown	19.1	4.66 (3.37‐6.44)	<.001	1.29 (0.91‐1.86)	.157
LN metastases					
Yes	44.7	1.00		1.00	
No	36.3	1.16 (0.98‐1.38)	.089	0.84 (0.70‐1.01)	.066
Unknown	12.2	2.03 (1.59‐2.59)	<.001	0.94 (0.72‐1.23)	.641
Differentiation					
Well differentiated	59.0	1.00		1.00	
Moderately differentiated	48.3	1.32 (1.01‐1.73)	.039	1.18 (0.90‐1.55)	.225
Poorly differentiated	7.2	8.33 (6.82‐10.2)	<.001	4.48 (3.57‐5.62)	<.001
Undifferentiated	7.9	8.16 (6.43‐10.36)	<.001	4.17 (3.20‐5.44)	<.001
Primary tumor resection					
No	15.4	1.00		1.00	
Yes	57.0	0.31 (0.26‐0.36)	<.001	0.48 (0.39‐0.59)	<.001

Note

Other*: American Indian/AK Native, Asian/Pacific Islander, unknown.

Abbreviations: LN: lymph nodes; CI: Confidence interval; OS: Overall survival; HR: Hazard ratio.

### 3.3 Survival analysis in the PTR subgroup

After excluding the patients whose primary tumor size was unknown, a total of 866 patients were categorized into the PTR subgroup. As shown in Table 4, univariate and multivariate analyses were performed to analyze the prognostic risk factors for survival. The results suggested that old age (≥60 years), a large primary tumor (≥4 cm), and poor tumor differentiation (poorly differentiated and undifferentiated) independently increased the risk of death. By contrast, we observed that primary tumors that originated from the small intestine (HR = 0.31, 95% CI: 0.14‐0.67, *P* = .003) and pancreas (HR = 0.28, 95% CI: 0.12‐0.63, *P* = .002) showed better survival than those from the stomach and colorectum.

**Table 4:** Univariate and multivariate survival analyses of overall survival in patients who underwent PTR (N = 866)

Variables	5‐year OS (%)	Univariate analysis		Multivariate analysis	
		HR (95% CI)	*P* value	HR (95% CI)	*P* value
Gender					
Male	55.2	1.00		1.00	
Female	59.3	0.88 (0.68‐1.14)	.327	0.86 (0.66‐1.11)	.241
Age at diagnosis					
<60	63.7	1.00		1.00	
≥60	49.3	1.69 (1.31‐2.18)	<.001	1.70 (1.32‐2.20)	<.001
Primary tumor site					
Stomach	39.7	1.00		1.00	
Small intestine	73.3	0.27 (0.13‐0.59)	<.001	0.31 (0.14‐0.67)	.003
Colorectum	24.6	1.86 (0.87‐3.97)	.110	0.80 (0.37‐1.74)	.572
Pancreas	59.7	0.44 (0.20‐0.99)	.047	0.28 (0.12‐0.63)	.002
Primary tumor size*					
≤2 cm	73.1	1.00		1.00	
2‐4 cm	62.0	1.72 (1.14‐2.59)	.010	1.34 (0.88‐2.04)	.167
≥4 cm	39.7	4.12 (2.82‐6.03)	<.001	1.91 (1.25‐2.93)	.003
Differentiation					
Well differentiated	71.1	1.00		1.00	
Moderately differentiated	54.7	1.48 (0.99‐2.19)	.050	1.32 (0.89‐1.95)	.176
Poorly differentiated	17.4	9.87 (7.14‐13.65)	<.001	5.47 (3.73‐8.02)	<.001
Undifferentiated	19.4	11.67 (8.02‐16.98)	<.001	5.11 (3.29‐7.91)	<.001

Note

Primary tumor size*: patients with unknown size were excluded.

Abbreviations: LN: lymph nodes; CI: Confidence interval; OS: Overall survival; HR: Hazard ratio.

## 4 DISCUSSION

There is still controversy regarding the surgical treatment of metastatic NENs due to their relatively heterogeneous behavior and the lack of clinical evidence. In our study, PTR was identified as an independent prognostic factor of survival in the multivariate analysis. Our results support the hypothesis that resection of the primary tumor is associated with favorable survival in GEP‐NEN patients with LM and should be considered in selected patients. To our knowledge, the current study is the largest retrospective cohort study to evaluate the survival benefit of PTR among GEP‐NEN patients without undergoing liver surgery.

To achieve curative intent, the complete removal of primary and metastatic tumors should be considered. Earlier studies have found that PTR improved the 5‐year OS rates of patients, ranging from 33.3% to 74.0%, irrespective of tumor function.^9^, ^10^ Consistent with these results, compared to the nonresection group, an improvement in 5‐year OS rates (from 15.4% to 57.0%) was observed in our study.

In the PTR subgroup, we found that patients with primary tumors originating from the small intestine and pancreas had a better prognosis than those with primary tumors originating from the colorectum and stomach. Notably, patients with colorectal NENs had the worst OS than any other subtype. Previous studies have shown a 5‐year survival rate of 15%‐30% in colorectal NENs with distant metastases, which was consistent with the lowest survival rate that we reported.^11^, ^12^ In contrast, patients with small intestinal NENs who underwent PTR had relatively better survival, with 5‐year OS rates of 73.3%, which compared well with a recently published systematic review that showed improved 5‐year OS rates (from 36.6% to 73.1%).^13^ It is worth noting that 80.4% (447/556) of patients underwent PTR in this subtype. The rationale behind the high percentage of resection is likely because of symptomatic reasons.^14^ Many patients presented with clinical complications, such as flushing or diarrhea caused by excess hormones and obstruction and malnutrition due to large local tumors. Even if metastatic disease exists, the European Neuroendocrine Tumor Society (ENETS) guidelines recommend that resection be considered for these patients to relieve symptoms or for patients in whom obstruction may occur in the future.^15^ Interestingly, a study by Kosmas et al demonstrated that prophylactic surgery for primary tumors compared to no surgery or delayed surgery may not show any favorable benefit in asymptomatic patients with LM.^16^ However, in this study, more than half of the patients in the delayed group eventually underwent surgery, which might impact survival results. Although symptomatic information was not available from the SEER database, there is no doubt that PTR for symptomatic patients with LM is highly recommended.

Gastric NENs with LM also had a worse prognosis despite undergoing PTR. The main reason for this finding was probably that 65.6% (65/99) of gastric NENs were identified as poorly differentiated or undifferentiated in our study. Gastric NENs in type II and type III are mostly large lesions with a high metastasis rate (range from 10% to 100%) and low differentiation; however, we did not extract specific classifications from the SEER database.^17^ A study by John et al consisting of 983 patients with stage IV gastric NENs found that 114 patients who underwent PTR had prolonged survival compared to those who did not (21.2 months vs 7.0 months, respectively, *P* < .001).^7^ However, in Lewis's study, there was no survival advantage found in gastric NEN patients with LM who underwent PTR without liver treatment.^18^ Presumably, the different results in these studies were caused by the small sample size and selection bias. Additionally, the heterogeneous behavior of gastric NENs when they metastasize to the liver and whether or not they cause worse survival among different subtypes could not be ignored. More studies are needed to validate these results.

With regard to pancreatic NENs with LM, a prolonged 5‐year OS rate of 59.7% was comparable to previous studies whose 5‐year OS benefit ranged from 47.6% to 81%.^19^, ^20^ Because of their anatomical location, PTR is not as frequently performed in the pancreas, as shown in Table 2. At the same time, the prognostic significance of various tumor locations in the pancreas might also indicate different results. The study by Xavier et al reported prolonged survival in patients with primary tumors in the body or tail of the pancreas than those with tumors in the head (HR = 0.78, 95% CI: 0.65‐0.94, *P* = .0095).^21^ In particular, a complication rate of 29.6% after pancreaticoduodenectomy was reported in a study by Jillian et al, which might be higher than that for central and distal pancreatectomy.^22^ Given this finding, some authors recommend that PTR be carried out in tertiary, high‐volume hospitals to minimize postoperative morbidity and mortality.^23^

Several reasons may explain why PTR could benefit patient survival. First, PTR may be performed for palliative and prognostic aims, because it not only reduces the tumor burden but also controls carcinoid syndrome and local tumor‐related symptoms. Second, PTR, to some degree, could delay the progression of LM. Liver failure is the most common cause of death. A study by Babak et al suggested that PTR could remove the source of LM and reduce essential hormones or growth factors that stimulate tumor proliferation, which finally translated into significantly prolonged survival.^20^ Moreover, according to a study by Emilio et al, PTR might enhance the efficacy of peptide receptor radionuclide therapy (PRRT) against pancreatic NENs with LM, prolonging the median OS from 65 months to 112 months (*P* = .011) and the median progression‐free survival from 30 to 70 months (*P* = .002).^24^ Based on previous data, the resection of LM has been proven to be associated with a highly prolonged survival time.^25^ In addition, the efficacy of several promising treatment options, such as transarterial chemoembolization, systemic chemotherapy, PRRT, somatostatin analogs, and liver transplantation, has been proposed for liver metastatic NENs.^3^ From this perspective, the value of a multimodal approach of combining PTR with adjuvant therapies or liver‐directed treatments should be investigated in the future.

The secondary aim of this study was to identify predictive factors for selecting patients who might benefit from PTR. As shown in Table 4, the multivariate analysis suggested that the surgical treatment of patients older than 60 years, with a primary tumor larger than 4 cm, and a tumor with low differentiation be considered carefully if no fatal symptoms occur, because these variables increased the risk of death. Notably, although PTR might prolong the survival time in patients with poorly differentiated and undifferentiated tumors, we do not recommend surgery for this group of patients because of the low survival benefit shown in our study, which is probably due to high recurrence after PTR.^26^

Although we found that LN metastases were strongly associated with patients who received PTR (Table 2), the possibility of patients who underwent PTR resulting in a greater chance of detecting positive LN metastases could not be ruled out. On the other hand, the multivariate analysis demonstrated that LN metastases were not an independent prognostic factor for patients (Table 3). A similar result was also found in Peng's study, and they demonstrated that an LN‐positive ratio greater than 0.4 was an independent risk factor for pancreatic NENs.^27^ Therefore, the LN‐positive ratio may be a strong predictive factor for identifying high‐risk patients.

There are some limitations to our study. First, it was a retrospective study that was probably affected by selection bias. However, a prospective study is difficult to conduct because of multiple reasons, such as the rare incidence, consent of patients, and financial status. Second, information on tumor burden, the Ki‐67 index, and chemotherapy treatment were not available in the SEER database; therefore, their effects on prognosis could not be analyzed in this series.

## 5 CONCLUSION

Despite these limitations, we believe that the samples and follow‐up time were enough to support our findings. Our study revealed that resection of the primary tumor is an independent prognostic factor that prolonged OS in all GEP‐NEN patients with LM using the SEER database. However, poor survival in patients who undergo PTR may be associated with an age older than 60 years, a primary tumor larger than 4 cm, a tumor located in the stomach or colorectum, and poor differentiation. This finding may provide meaningful insights for surgeons to identify high‐risk patients.
